# Athens multifamily therapy after a first psychotic episode: Online therapy during the COVID-19 pandemic

**DOI:** 10.1192/j.eurpsy.2022.1976

**Published:** 2022-09-01

**Authors:** M. Selakovic

**Affiliations:** 1 National and Kapodistrian University of Athens Medical School, Eginition Hospital, First Department Of Psychiatry, Athens, Greece; 2 GH “Sismanogleio”, Department Of Psychiatry, ATHENS MELISSIA, Greece; 3 National and Kapodistrian University of Athens Medical School, Eginition Hospital, First Department Of Psychiatry, Athens, Greece

**Keywords:** multifamily therapy, online therapy, FEP, COVID-19 pandemic

## Abstract

**Introduction:**

The Athens Multifamily Therapy Project (A- MFTP) aims to provide systemic multifamily group therapy to youths who experienced a first psychotic episode (FEP) and their families

**Objectives:**

Since 2017, we run five groups of five-four families, with a duration of ten months and frequency every two weeks. Participants were recruited from the longitudinal study, Athens FEP Project, which aimed to investigate the involvement of genetic and environmental determinants on psychosis risk.

**Methods:**

During the Covid-19 pandemic, the provision of therapy to the current groups continued through online sessions. Participants were asked to answer qualitative questions on the perceived effectiveness of the therapy on their life as well as on the presenting problem(s) at three time points: middle, end of therapy and 6-month follow-up.

**Results:**

All members highlighted the significance of the reciprocity in the group communication. They mentioned that “sharing” and “exchanging” experiences helped them listen to others and felt heard by them. They moved from feeling fear and embarrassment when discussing the diagnosis and the aftermath, to feeling safety and comfort talking about their difficulties. Qualitative analysis showed no difference in participants’ perception of multifamily therapy as helpful between live therapy and online therapy.

**Conclusions:**

Results suggest that MFT can be a viable way to provide early intervention in FEP even in at online modality.

**Disclosure:**

No significant relationships.

